# Acrylic-based occlusal device materials – the influence of manufacturing techniques on material properties and the propensity for biofilm formation

**DOI:** 10.2340/biid.v13.45909

**Published:** 2026-04-24

**Authors:** Ketil H. Haugli, Jan Tore Samuelsen, Vigdis Aas, Inger S. Dragland, Colin Charnock

**Affiliations:** a Department of Life Sciences and Health, Oslo Metropolitan University, Oslo, Norway; b Nordic Institute of Dental Materials (NIOM), Oslo, Norway

**Keywords:** CAD/CAM, material characteristics, dental biofilm

## Abstract

**Objective:**

The material composition, manufacturing system, and post-processing steps used to fabricate acrylic-based occlusal devices may affect their clinical performance. This study aims to assess how manufacturing techniques and post-processing treatments influence the material properties of acrylic-based occlusal devices and the propensity of *Streptococcus mutans* to form biofilms on the surfaces.

**Materials and methods:**

Based on applied manufacturing technique and post-processing treatment, disc‑shaped specimens were manufactured using four 3D printing workflows (Splint 2.0‑Asiga Flash/Otoflash (OF), and LT Clear‑Form Cure/OF), one milling workflow (Therapon), and one autopolymerization workflow (PalaXtreme). Water sorption and solubility, surface free energy (SFE), average surface roughness, and Vickers hardness were tested across these workflows. The ATCC 700610 *Streptococcus mutans* strain served as a model for biofilm formation on the material surfaces. Two biofilm methods were employed: a 24-hour bioreactor approach and a 72-hour culture plate approach. Biofilm was quantified as colony-forming units per cm^2^.

**Results and conclusion:**

The Therapon and PalaXtreme workflows exhibited the lowest solubility, suggesting that these materials have the lowest release of material components in water. The Splint 2.0 workflows exhibited the lowest water sorption, indicating enhanced material integrity in humid conditions. Therapon showed the highest Vickers hardness, followed by PalaXtreme. The lower hardness of the print materials may make them susceptible to wear, which may not be optimal for treating patients with bruxism. No significant differences in SFE were observed between workflows. Low roughness values across all workflows indicate good polishability, which can enhance resistance to bacterial adhesion. In the 72-hour biofilm experiment, the Therapon workflow exhibited the most biofilm formation on material surfaces while PalaXtreme showed the least (*p* < 0.05). No significant differences between workflow groups were shown in the 24-hour biofilm experiment.

In summary, material properties are influenced by material chemistry, manufacturing method, and associated post-processing treatment.

## Introduction

Acrylic-based occlusal devices, also known as dental splints, have several applications in dentistry. These devices are commonly used in orthodontics to treat patients with temporomandibular disorders (TMD) and bruxism, or to facilitate dental stability or alignment [1, 2]. In managing bruxism, prolonged use of occlusal devices is usually recommended, and exposure to high occlusal loads can be expected, which can affect their clinical performance [3].

Manufacturers of custom-made oral and dental appliances are increasingly adopting computer-aided design and manufacturing (CAD/CAM) to meet quality requirements and remain competitive in an efficiency-driven market [4]. Computer numerical control (CNC) milling machines are a well-established manufacturing method, widely recognized for their high precision, consistency, and reliability [5, 6]. Recently, three-dimensional (3D) printing has gained attention as an alternative method for producing dental and oral appliances [7]. In general, 3D printing offers advantages such as design versatility, enabling the fabrication of complex geometries that may be unachievable with conventional milling. Additionally, it can offer improved cost-effectiveness and reduce material waste, overcoming certain milling challenges [8]. However, several variables can influence the quality of acrylic-based occlusal devices produced through 3D printing. These variables include the choice of vat photopolymerization technologies, such as stereolithography (SLA) and digital light processing (DLP), as well as printing parameters, including layer thickness, support structures, and object orientation on the build plate. Additionally, post-processing treatments, including rinsing, post-curing conditions, and surface finishing, can vary widely. These variables make it challenging to establish consistent workflow routines and standards needed to ensure safe and reliable production of acrylic-based occlusal devices [9]. The transition to digital production has been considered a paradigm shift in dentistry [10]. However, there is currently a lack of evidence regarding the efficacy of CAD/CAM-produced oral appliances, and limited documentation of the impact of CAD/CAM manufacturing techniques on the biological properties of acrylic-based occlusal devices [11–14].

The highly variable and harsh oral environment makes oral biomaterials susceptible to degradation and wear, which affects their material integrity, strength, and biocompatibility. These challenges generally include mechanical stresses from mastication, fluctuations in temperature and salivary pH, and exposure to diverse enzymatic and microbiological communities [15, 16]. Saliva contains approximately 99% water [17]. Therefore, assessing water sorption and solubility resistance is a clinically relevant measure of a material’s suitability for use in the oral environment. A standardized water sorption and solubility test for orthodontic base polymers is described in ISO 20795-2 [18] and is used in this study. Preferably, both parameters should be as low as possible, since water sorption affects material stability and integrity, whereas solubility is directly linked to material loss due to dissolution in water. Such a release may further affect material biocompatibility. The hardness values of dental biomaterials provide information on their ability to resist localized plastic deformation under indentation by a harder substance. Harder substances or materials can abrade softer materials, thus making the less hard acrylic-based materials more susceptible to abrasion by tooth enamel or restorative materials [19]. Consequently, there is an increased risk of patient exposure to chemicals and microplastic particles from occlusal devices with low hardness values. Material wear may also promote scratches and roughen affected areas, creating more sites for bacterial adherence and growth [20]. Current literature provides limited evidence on how digital manufacturing methods influence microbial adhesion and growth on acrylic-based occlusal device surfaces [14].

Comprising the second-most diverse microbiome after the gut, the oral cavity hosts more than 700 bacterial species, as well as fungi, viruses, and protozoa [21]. The oral microbiome colonizes the surfaces of hard dental tissue and soft gingival and mucosal tissue in organized communities within a healthy oral cavity and has been proposed to be site- and subject-specific [22]. Furthermore, within minutes after cleaning, both hard and soft oral tissues are covered by a film of salivary glycoproteins, forming the acquired pellicle, which is believed to play a vital role in the initial attachment and colonization of bacteria [23]. The soft oral lining epithelium sheds at least three times daily, and this is believed to be an effective mechanism for reducing bacterial adhesion [24]. Teeth and dental biomaterials, on the other hand, exhibit non-shedding surfaces, making them more susceptible to microbial colonization and biofilm formation. When biomaterials are introduced into the oral cavity, the healthy oral microbiome may be challenged due to the establishment and growth of pathogenic microorganisms, such as *Streptococcus mutans* and *Candida albicans.* These microorganisms are involved in the oral pathogenesis of dental caries, periodontitis, peri-implantitis, denture stomatitis, and candidiasis [25–27].

Bacterial adhesion and biofilm maturation are governed by a multifactorial interplay between the physicochemical properties of the bacterial cell surface and the material substrate, encompassing surface chemistry, surface free energy (SFE), surface charge, and topographical features [24, 28–31]. Increased surface roughness of the material consequently increases surface area, potentially providing more binding sites for bacterial attachment and growth. An irregular surface facilitates bacterial attachment in small pits and crevices, exposing a greater portion of the cell surface to the substratum while shielding bacteria from shear forces. Generally, high-SFE substrates have a greater propensity for microbial adhesion than low-SFE substrates; however, surface roughness is considered the more predominant factor over SFE [32].

This study aimed to compare selected acrylic-based occlusal device materials from different manufacturing techniques. In addition to conventional autopolymerization and milling, we included two different 3D printing technologies. For the 3D-print materials, we also compared various post-curing protocols. Chemical, physicochemical, and mechanical properties, as well as biofilm-forming propensity, were selected for comparison between the materials.

## Materials and methods

### Overview of materials and manufacturing technology

The acrylic-based occlusal device materials were manufactured by two vat photopolymerization 3D printing technologies, CNC milling, and an autopolymerization powder/liquid system. Table 1 presents an overview of the selected acrylic-based occlusal device materials and the manufacturing technology or system used to produce the material specimens. Additionally, the manufacturers’ listed compositional details for the materials are provided.

**Table 1:** Information on the selected materials and their chemical compositions, as provided by the manufacturers.

Material, manufacturer and lot	Manufacturing technology/system	Composition as stated in material data safety sheet (quantity in %)
**Freeprint Splint 2.0** (DETAX, Ettlingen, Germany LOT: 220807)	Print – Digital Light Processing (DLP) **Asiga MAX UV** (Asiga, Sydney, Australia)	Isopropylidenediphenol PEG-2 dimethacrylate (Bis-EMA) (90 – < 95) 2-Propenoic acid, (5-ethyl-1,3-dioxan-5-yl)methyl ester (1 – < 5) Diphenyl(2,4,6-trimethylbenzoyl)phosphine oxide (TPO) (1 – <5)
**Dental LT Clear V2** (Formlabs, Somerville, MA, USA LOT: DC01220622-02)	Print – Stereolithography (SLA) **Form 2** (Formlabs, Somerville, MA, USA)	Bisphenol A dimethacrylate (50 – 70) Urethane dimethacrylate (25 – 45) 2-Hydroxyethyl methacrylate (7 – 10)
**Therapon Transpa** (Zirkonzahn, Gais, Italy LOT: 14058)	Mill – Computer numerical control (CNC) **M1 Wet Heavy Milling unit** (Zirkonzahn, Gais, Italy)	Acrylic polymethylmethacrylate copolymer
**PalaXtreme** (Kulzer, Hanau, Germany LOT: K010021)	Autopolymerization **Powder/liquid system**	**Powder** 1-Benzyl-5-phenylbarbituric acid (0 – 5) Methyl methacrylate (≥ 0.1 – <1) 2-ethylhexyl thioglycolate (≥ 0.1 – <1) Dibenzoyl peroxide (≥ 0.1 – <1) **Liquid** Methyl methacrylate (75 – 90) 2-[[(Butylamino)carbonyl]oxy]ethyl acrylate (5 – 10) Aliphatic urethane acrylate (0 – 5) (2,4,6-Trioxo-1,3,5-triazinane-1,3,5-triyl) triethylene triacrylate (≥ 1 – < 2.5) Quaternary ammonium compounds, tri-C8–C10-alkylmethyl chlorides (≥ 0.025 – < 0.25)

### Overview of the defined workflows and associated post-processing treatments

Predefined workflows for the manufacturing of acrylic-based materials were established based on the manufacturing technique and associated post-processing treatments. Table 2 presents the six defined material workflows and their respective post-processing treatments. According to the manufacturer’s post-curing protocols, the Otoflash (OF) G171 (NK Optik, Baierbrunn, Germany) unit is validated for the FREEPRINT Splint 2.0 material [33], whereas the Form Cure (FC) (Formlabs, Somerville, MA, USA) unit is validated for the Dental LT Clear V2 material [34]. The alternative post-curing methods in this study should be regarded as experimental.

**Table 2:** Information on the selected materials and corresponding workflows and post-processing treatments.

Workflows	Post-processing treatments					
	Rinse	UV-curing			Grind and polish	dH_2_O storage
		Asiga flash (AF)	Form cure (FC)	Otoflash G171 (OF)		
DLP – Print: **Splint 2.0 – AF**	Isopropanol (IPA). 2 × 3 minutes in ultrasonic bath 5 minutes in fresh IPA	15 minutes × 2			Wet SiC FEPA P: 1200, 2400, 4000	No
DLP – Print: **Splint 2.0 – OF**	IPA 2 × 3 minutes in ultrasonic bath 5 minutes in fresh IPA			2000 flashes × 2. With nitrogen gas	Wet SiC FEPA P: 1200, 2400, 4000	No
SLA – Print: **LT Clear – FC**	Form Wash IPA for 15 minutes 5 minutes in fresh IPA		60 minutes 60°C		Wet SiC FEPA P: 1200, 2400, 4000	No
SLA – Print: **LT Clear – OF**	Form Wash IPA for 15 minutes 5 minutes in fresh IPA			2000 flashes × 2. With nitrogen gas	Wet SiC FEPA P: 1200, 2400, 4000	No
Milling: **Therapon**	–	–			Wet SiC FEPA P: 1200, 2400, 4000 grit	No
Autopolymerization: **PalaXtreme**	–	–			Wet SiC FEPA P: 1200, 2400, 4000 grit	Yes 12 hours

The rows depict the six workflows in the study design: Four for printing, one for milling, and one for autopolymerization. DLP: Digital Light Processing; SLA: stereolithography.

### Specimen design and dimensions

All the prepared specimens were disc-shaped (Online Supplementary Figure S1). The 3D-printed specimens were printed perpendicular to the build plate (90°) using the maximum available z‑axis resolution for each material (50 µm for Splint 2.0 and 100 µm for LT Clear). For the milling material, cylindrical specimens were CNC milled (M1 Wet Heavy Milling unit (Zirkonzahn, Gais, Italy) from the Therapon Transpa block and subsequently sectioned to the desired dimensions under continuous water cooling using a precision cutting machine (Secotom-60; Struers, Ballerup, Denmark). The autopolymerized specimens were processed in accordance with the manufacturer’s instructions (20 g powder:12 g liquid; cured at 55°C for 40 min under 2 bar pressure). For the SFE, surface roughness, Vickers hardness test, including the 72-hour culture plate approach, the specimens were prepared with dimensions *h* = 2.0 mm and *d* = 10.0 mm. In the 24-hour bioreactor approach, specimens were prepared to fit the rods for specimen mounting in the bioreactor (*h* = 3.0, *d* = 12.5 mm). Specimens for the sorption and solubility test were prepared to *h* = 0.5 mm and *d* = 50 mm, following ISO 20795-2:2013 [18]. However, this standard does not include instructions for digital production. Due to feasibility constraints, the printed specimens were fabricated directly on the build plate (i.e. oriented parallel to it), one at a time. Specimens for the milling material were manually manufactured by a qualified toolmaker. Briefly, cylindrical specimens were initially cut out of the milling block using a hole saw (55 in diameter). The cylinders were then mounted in a lathe (WF290V‑F; LTS Maskin AS, Lonevåg, Norway) and adjusted to a final diameter of 50 mm. Discs were subsequently sectioned from each cylinder using the Secotom-60 precision cutting machine (as previously described). The discs were finally mounted in a manual milling machine (FTV‑2, Lagun, Spain), and fine milling was performed to achieve a uniform thickness of 0.50 mm for each specimen.

### Water sorption and solubility

Water sorption and solubility tests were conducted in accordance with ISO 20795-2:2013 for orthodontic base polymers [18]. For the sorption and solubility experiments, five independently fabricated specimens were tested per workflow (*n* = 5). The specimens were placed in a desiccator containing dry silica gel and stored at 37 ± 1°C for 23 ± 1 hour. Then, the specimens were transferred to a second desiccator with dried silica gel for 1 hour prior to weighing with an analytical balance (Mettler AE 163; Mettler-Toledo Inc., Columbus, OH, USA). This process was repeated every 24 hours until the weight difference between successive measurements was less than 0.2 mg, establishing the baseline mass (m_1_). The volume (V) of each specimen was calculated using its diameter and thickness measurements taken with a digital caliper (Mahr 16 EX; Mahr, Göttingen, Germany). Next, the specimens were immersed in Grade II water at 37 ± 1°C for 7 days. After immersion, each specimen was carefully removed with a polymer-coated tweezer, surface-dried according to the ISO standard, and weighed to determine the mass (m_2_). A reconditioning desorption process was then carried out by placing the specimens in a desiccator containing fresh, dry silica gel at 37 ± 2°C for 1 d, followed by transferring them to a second desiccator with freshly dried silica gel for 1 hour at room temperature. Reweighing continued until the weight difference between successive measurements was less than or equal to 0.2 mg, indicating a constant reconditioned mass (m_3_). Finally, the water sorption (W_sp_) and solubility (W_sl_) were calculated using equations I and II, respectively, and expressed in µg/mm^3^.

I: W_sp_ = (m_2_ – m_3_) / V

II: W_sl_ = (m_1_ – m_3_) / V

### Vickers hardness

Vickers hardness was measured using a Duramin‑40 hardness tester (Struers, Ballerup, Denmark), operated according to the manufacturer’s built-in protocol for the Vickers 500 g microhardness test. A pyramid-shaped diamond indenter with a square base was pressed vertically onto the material surfaces with a 500 g load and a 10-second dwell time. This was performed at five different locations on each specimen and the mean of the five recordings was reported as one datapoint. Three independent specimens (*n* = 3) from each workflow were tested. The diagonals of the square indentation were measured and used in the Vickers hardness calculations. Mean values for each workflow were reported as HV 0.5.

### Surface free energy

To determine the SFE of the materials, the sessile drop method protocol for the optical contact angle instrument DSA 30 standard (Krüss, Hamburg Germany) was used. Three independent specimens from each workflow were selected (*n* = 3). The specimens were wet-polished (up to 4000 FEPA P grit size), ultrasonically cleaned in distilled water for 10 minutes, and then dried in a desiccator with fresh silica gel stored in a 37°C chamber for 24 hours. The experiments were conducted at room temperature (23 ± 2°C). An automatic dosing system delivered 2 µL drops of high-purity Millipore water and diiodomethane (Sigma-Aldrich, St. Louis, MO, USA) as reference liquids. The contact angle was measured 10 seconds after drop placement, and 10 recordings were taken at 5-second intervals. This procedure was repeated three times on predefined locations (left, right, and center) on each specimen surface with each reference liquid. Data analysis and reporting were performed using the instrument software (ADVANCE ver. 1.16.1.33401, Krüss, Hamburg, Germany). The baseline was set at the three-phase point interface involving the liquid, air, and solid surface. The SFE of the acrylic-based specimens was calculated using the Owens, Wendt, Rabel, and Kaelble (OWRK) model (Online supplement) [35, 36], which requires polar (water) and non-polar (diiodomethane) liquids with known surface tension values (at 20°C). Results were reported in mJ/m².

### Surface roughness

Average roughness (Ra) values for the workflows were obtained using a surface roughness tester (Surftest SJ-201P; Mitutoyo Scandinavia AB, Upplands Väsby, Sweden). Surface roughness measurements on the acrylic specimens were performed by following the manufacturer’s operating specifications for the instrument. Additionally, non-ground and polished specimens from the print materials were measured to assess potential differences in roughness after printing. The average of five measurements taken at predefined locations on the surface (upper, lower, left, right, and center) of each specimen (technical replicate) was recorded. This process was repeated for three independent specimens (*n* = 3) from each workflow group. The evaluation length was set to 2.5 mm. Mean Ra values and standard deviations (SD) were reported in µm.

### The biofilm experiments

The acrylic specimens were disinfected for 10 minutes in 70% ethanol and washed three times with sterile distilled water.

Two methods were used to study biofilm formation: (1) using a Centers for Disease Control and Prevention (CDC) biofilm reactor (BioSurface Technologies, Corp., Bozeman, UT, USA) (Online Supplement Figure S2) to grow biofilm for 24 hours (‘24-h bioreactor approach’), and (2) cultivating biofilm on surfaces by submerging specimens in 12-well culture dishes with inoculated growth medium for 72 hours (‘72-h culture plate approach’). Due to the lack of standardized methods for both approaches, the feasibility of the experiments was assessed by two microbiologists. The adhesion test described by He et al. [37] was partly adopted and served as a basis for the 72-hour culture plate approach used in this study. In both experiments, the wild‑type Streptococcus mutans UA159 strain (ATCC 700610) was used as the inoculum. This was grown prior to use as inoculum on brain heart infusion (BHI) agar either in a 5% CO_2_ atmosphere at 37°C for 48 hours (24-hour bioreactor approach) or anaerobically (72-hour culture plate approach).

In the 24-hour bioreactor approach, 2–3 colonies from the agar plate were inoculated into 10 mL of BHI broth and incubated for 24 hours in an incubator with 5% CO_2_ at 37°C. The overnight culture was then measured with a PV4 Visible spectrophotometer (VWR, Darmstadt, Germany) at OD600 nm, and the turbidity was adjusted to OD = 0.80 by subtracting the blank to set the initial cell concentration. The inoculum was transferred to BHI broth containing 1% sucrose at a 1:100 dilution. Next, 340 mL of the inoculated medium was transferred to the biofilm reactor. Two specimens from each workflow were mounted vertically in the bioreactor’s specimen holder and immersed in the inoculated medium. A magnetically driven stir bar (100 rpm) maintained a steady dynamic environment in the growth medium. The bioreactor was placed in an incubator set at 37°C with 5% CO_2_ for 24 hours. Subsequently, the specimens were carefully removed from the holder, washed three times with phosphate-buffered saline (PBS) (Lonza, Walkersville, USA), and transferred to sealed tubes containing 2 mL PBS and glass beads (*d* = 3 mm). The tubes were shaken for 30 seconds using a vortex shaker (VWR, Darmstadt, Germany) before performing serial dilutions in PBS. Then, triplicate 25 µL droplets were placed on BHI agar plates, and re-incubated for about 24 hours under the same incubation conditions. Finally, colony-forming units (CFU) were counted, and biofilm results were reported as CFU/cm^2^ for each workflow.

In the 72-hour culture plate approach, colonies were transferred from the agar plate to prepare an inoculum corresponding to a McFarland 0.5 density, as determined spectrophotometrically (DEN-1 McFarland Densitometer; Biosan, Riga, Latvia). The inoculum was diluted 1/1000 in BHI broth containing 1% sucrose. Two mL of the inoculum was added to disc specimens (one per well) in a 12-well culture plate. The plates were placed in an anaerobic chamber containing an anaerobic sachet (Oxoid AnaeroGen 3.5 L; Thermo Fisher Scientific, Basingstoke, UK) and incubated for 72 hours at 37°C. After 48 hours, the growth medium and the anaerobic sachets in the vessels were replaced with fresh medium and sachets. Then, after a further 24 hours of incubation, the specimens were carefully removed with tweezers, washed 3 times with PBS, and dried for 5 minutes in a biological safety cabinet under aseptic conditions. Thereafter, the specimens were transferred to individual wells in 12-well plates, and 2 mL of Maximum Recovery Diluent (MRD) containing 0.01% Tween was added to each well. All surfaces of the specimens were thoroughly scraped using a single 1.5 mm dental microbrush applicator per well to maximize biofilm recovery and reduce inter-sample variability. The duration and pattern of scraping were carefully standardized across all specimens. The suspensions and the tips of each respective brush were transferred to individual Eppendorf tubes containing acid-washed, sterile glass beads (0.5 mm) and immediately put on ice. Each tube was vortexed three times for 30 s with intermittent cooling. A dilution series in MRD was performed before applying the droplets to the BHI agar plates for subsequent CFU counting, as described for the 24-hour bioreactor approach. The agar cultures were incubated for 24–36 hours under anaerobic conditions at 37°C, and colonies were counted. Lastly, CFU were counted and reported as CFU/cm^2^ for each workflow.

For both biofilm approaches, three independent experiments were performed, with two independent specimens (*n* = 2) per workflow group per experiment (including negative controls). The positive culture medium control was microscopically evaluated for microbial growth.

### Statistics

The study employed a multi‑factor experimental design in which the primary independent variable was the material workflow group (i.e. the different manufacturing methods and associated post-processing treatments applied for the occlusal device specimens). The dependent variables were the measured material and biological response outcomes, including water sorption and solubility, SFE, surface roughness (Ra), Vickers hardness test (HV 0.5), and biofilm formation. Each dependent variable was quantitatively assessed for all workflow groups to determine the effect of manufacturing on material properties and biofilm behavior.

Data analyses and graph visualizations were performed using GraphPad Prism (Version 10.4.1; GraphPad Software, San Diego, CA, USA). For the SFE, surface roughness analyses, Vickers hardness test, and biofilm experiments, the normality of the datasets was assessed using the Shapiro-Wilk test. One-way analysis of variance (ANOVA) was conducted, followed by Tukey’s multiple comparisons test. The nonparametric Kruskal-Wallis test, followed by Dunn’s multiple comparison test, was performed for the sorption and solubility data, as normality was not confirmed. Statistical significance was set at α = 0.05; accordingly, *p*-values below 0.05 were considered statistically significant. An overview of all *p*-values is provided in the Online Supplementary Material.

## Results

### Sorption and solubility

**Figure 1:**
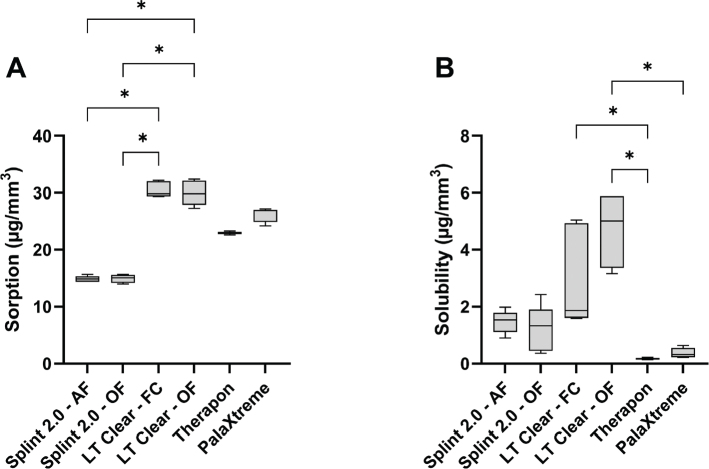
Sorption (A) and solubility (B) experiments of the different workflows. The box plots show the median, the lower 25th percentile, and the upper 75th percentile. Error bars indicate maximum and minimum values. Asterisks (*) indicate significant differences (*p* < 0.05).

Figure 1 summarizes the sorption and solubility measurements of the materials from the workflows. The water sorption test showed significant differences between the LT Clear and Splint 2.0 workflows, with Splint 2.0 exhibiting the lowest water sorption regardless of the applied post-curing protocol (Figure 1A). The choice of post-curing treatment did not affect the overall water sorption capacity of the respective print materials. The Therapon workflow, including PalaXtreme, showed the lowest water solubility, while the LT Clear – OF workflow had the highest.

### Hardness

**Figure 2:**
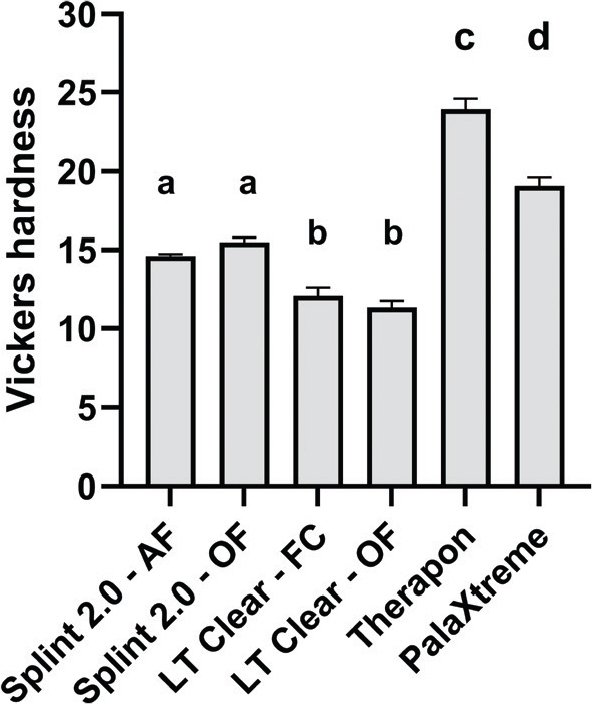
Overview of Vickers hardness results (HV 0.5) for the material workflows. Groups that do not share the same letter above the bar differ significantly (*p* < 0.05). Data are reported as mean, and error bars represent SD.

The Therapon workflow showed the highest mean HV 0.5 value (23.9), while the SLA print material showed the lowest mean HV 0.5 values (11.3 for the LT Clear-OF workflow and 12.1 for the LT Clear-FC workflow; Figure 2). All group comparisons were statistically significant (*p* < 0.05), except for the within-group comparisons of the respective print materials.

### Surface free energy

The total SFE, calculated as the sum of polar and dispersive components, ranged from 41.19 to 48.22 mJ/m² across the material workflows, with no statistically significant differences (*p* > 0.05) observed between the groups (Table 3). This lack of significance was also evident when comparing the polar (hydrophilic) and dispersive (hydrophobic) components.

**Table 3:** Measurements of the SFE of the workflows.

Workflow	Contact Angle (°)		SFE – Disperse (mJ/m^2^)	SFE – Polar (mJ/m^2^)	SFE – Total (mJ/m^2^)
	dH_2_O	Diiodomethane			
Splint 2.0 – AF	73.46 ± 0.60	38.13 ± 2.63	40.52 ± 1.29	5.70 ± 0.40	**46.22 ± 0.86**
Splint 2.0 – OF	72.94 ± 0.29	37.77 ± 6.59	40.60 ± 3.29	5.92 ± 0.78	**46.52 ± 2.33**
LT Clear – FC	70.86 ± 0.20	35.61 ± 2.11	41.74 ± 0.98	6.48 ± 0.14	**48.22 ± 0.81**
LT Clear – OF	70.28 ± 2.75	39.79 ± 3.31	39.69 ± 1.68	7.29 ± 0.62	**46.98 ± 2.44**
Therapon	76.51 ± 3.14	47.71 ± 11.73	35.37 ± 6.51	5.82 ± 0.66	**41.19 ± 5.94**
PalaXtreme	73.86 ± 1.67	39.43 ± 2.39	39.88 ± 1.20	5.71 ± 0.66	**45.59 ± 0.92**

The reference liquids dH_2_O and diiodomethane were used to calculate the polar and dispersive components of material surfaces of the respective workflows. Values are given as mean ± SD. SFE: surface free energy; AF: Asiga Flash; FC: Form Cure; OF: Otoflash.

### Surface roughness

All workflows showed low average surface roughness values, ranging from 0.08 µm (PalaXtreme) to 0.18 µm (Therapon), indicating that similar surface smoothness could be achieved regardless of the workflow used (Table 4). No significant differences in mean surface roughness were observed between the workflow groups (*p* > 0.05). However, the non-ground and polished print workflows exhibited higher surface roughness and greater variability, with mean values ranging from 2.09 to 4.52 µm. For these workflows, significant differences were observed when Splint 2.0 – Asiga Flash (AF) was compared with both LT Clear workflows (*p* < 0.05).

**Table 4:** The mean surface roughness (Ra) values for the workflows are shown, including Ra for specimens from the print workflows that were not modified by grinding and polishing.

Workflow	Ra	Ra without grind and polish
Splint 2.0 – AF	0.10 ± 0.04	2.09 ± 0.77*
Splint 2.0 – OF	0.13 ± 0.07	3.55 ± 0.97
LT Clear – FC	0.13 ± 0.07	4.52 ± 0.45
LT Clear – OF	0.12 ± 0.06	4.21 ± 0.49
Therapon	0.18 ± 0.14	–#
PalaXtreme	0.08 ± 0.05	–#

Values are reported in µm ± SD. AF: Asiga Flash; FC: Form Cure; OF: Otoflash.

* Indicates significant difference from LT Clear-FC and LT Clear-OF (*p* < 0.05).

# Not representative due to inconsistent parameters for reproducibility (bur wear, bur type, or mold material).

### Biofilm formation

**Figure 3:**
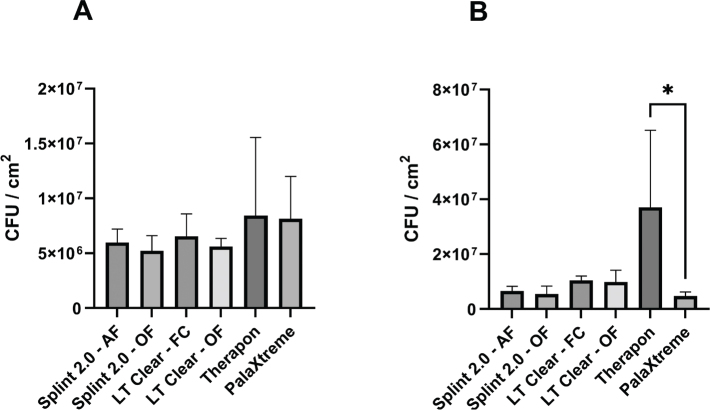
Quantification of *S. mutans* biofilm formation on surfaces of materials from the different workflows. The 24-hour bioreactor approach (A) and the 72-hour culture plate approach (B). Data are presented as mean and SD (error bars). Asterisk (*) indicates a statistically significant difference (*p* < 0.05).

Biofilm formed on all specimens, but no significant differences were observed with the 24-hour bioreactor approach (Figure 3A). In the 72-hour culture plate approach, a significant difference in biofilm formation (*p* < 0.05) was observed only when comparing the Therapon and PalaXtreme workflows (Figure 3B). Data are expressed as CFU/cm^2^.

## Discussion

The objective of the present study was to investigate the material properties of CAD/CAM-produced acrylic-based occlusal device materials. This study shows that different material compositions, manufacturing techniques, and post-processing treatments used to fabricate acrylic-based occlusal devices affect the final material properties. However, the complexity of modern acrylic-based materials, along with limited available information on their chemistry and composition due to trade secrets, makes it more difficult to predict and interpret their characteristics [38].

As saliva contains approximately 99.5% water, a materials water sorption and solubility properties are important aspects of dental biomaterials that are expected to affect their performance and longevity [39]. Water sorption and solubility should be kept as low as possible, as increased water sorption causes swelling that affects dimensional changes. Uptake of water molecules will spread between the polymer macromolecules, forcing them apart [40]. This has a softening effect due to the increased spacing between polymer chains, thereby reducing the materials’ mechanical properties. The ester-containing methacrylate groups in the polymer matrix make acrylic-based polymers prone to degradation in the oral environment due to hydrolytic activity, including bacterial acids and enzymatic activity [41]. Following ISO 20795-2:2013 [18], at least 4/5 tested specimens within a workflow group shall not exceed 32.0 µg/mm^3^ water sorption for the material to align with the standard.

Although the LT Clear workflows showed higher water-sorption values, all workflows complied with ISO 20795-2. There was a significant difference in water sorption between the print materials, with Splint 2.0 superior to LT Clear (Figure 1A). The post-curing treatment applied within the respective print materials did not affect their sorption capacity. This finding suggests that the difference in sorption compared to the other materials is likely due to material composition and/or structural inhomogeneities rather than the applied UV curing treatments. Regarding water solubility, to meet the standard requirements, 4/5 specimens must not experience a mass loss >5 µg/mm^3^ [18]. All workflows complied with the standard in this regard, except the LT Clear–OF workflow. According to the manufacturer, the OF G171 unit emits broad-spectrum, high-intensity light from two xenon lamps, totaling 200 W. In addition, nitrogen gas was introduced into the curing chamber to prevent oxygen inhibition, optimizing the post-curing conditions. The FC unit, on the other hand, uses 13 light-emitting diodes (LEDs; 405 nm), yielding a total output of 39 W. Although the long post-curing time of 60 minutes (at 60°C) might be beneficial for polymerization, higher post-curing temperatures up to 80°C have been shown to significantly increase the mechanical properties of SLA resins (thereby suggesting a higher degree of polymerization due to increased reaction kinetics) [42]. Nonetheless, the LT Clear workflows showed significantly higher solubility compared to the Therapon and PalaXtreme workflows. It might be expected that industrially produced milling blocks, such as Therapon Transpa, have improved properties due to better control over the polymerization process through temperature, pressure, and time. Nguyen et al. [43] investigated several mechanical properties of composite resin blocks produced by high-temperature/high-pressure polymerization (HT/HP) compared with those of photopolymerized resins. They reported increased hardness, fracture toughness, and Weibull modulus, as well as fewer and smaller voids in HT/HP resins compared with the photopolymerized counterpart. The authors attribute this to enhanced polymerization, reduced porosity, greater homogeneity, and reduced polymerization shrinkage during HT/HP processing of the resin blocks.

Increased temperature and pressure are also part of the polymerization protocol when fabricating occlusal devices using the autopolymerization (PalaXtreme) material [44]. The abovementioned treatments might explain the lower solubility of both Therapon and PalaXtreme workflows compared to the print materials. Since 3D printing involves successive layer-by-layer forming of a liquid into a solid, there is a risk of inhomogeneity in the printed object, which might affect the water sorption and solubility of these materials [45]. Due to a lack of pressure during polymerization, a less dense polymer network can be expected, potentially allowing water molecules to be absorbed between the polymer chains. On the contrary, the Splint 2.0 workflows exhibited the lowest water sorption. The high hydrophobicity of the bisphenol A ethoxylated dimethacrylate (bis-EMA) monomer can repel water, inhibiting its penetration into the material. The LT Clear material contains urethane dimethacrylate (UDMA) and, to some extent, 2-hydroxyethyl methacrylate (HEMA), which are incorporated into the resin matrix. The hydroxyl group of HEMA and the hydrogen bonds in the urethane backbone of UDMA may enhance the LT Clear material’s hydrophilic properties. The SFE results also showed that the LT Clear workflows had the highest polar components, which were only slightly higher than those of the other workflows. Thus, the elevated sorption and solubility values of the LT Clear material are likely multifactorial. Wulff et al. [46] did not observe a significant effect of the post-polymerization method on the water sorption of the tested 3D-printed resin-based splint materials, which aligns with our results. They suggested that material composition and build orientation are the more prominent factors influencing the water sorption and solubility of 3D-printed splint materials.

The Vickers Hardness test (HV 0.5) showed that the Therapon workflow exhibited the highest hardness value of 23.9. The results from the 3D printing materials suggest that the choice of post-curing method did not affect the materials’ hardness. AlRumaih and Gad [47] found that increased post-polymerization time and printing with layer thicknesses of 25 and 50 µm resulted in higher hardness than print materials printed at lower resolution (100 µm). Given that acrylic-based occlusal devices are commonly used to treat bruxism, careful consideration should be given to the material’s hardness when selecting the most suitable option for this patient group. Harder materials tend to offer greater wear resistance, suggesting that the Therapon and PalaXtreme workflows may be preferable choices. Given that the Vickers Hardness values of tooth enamel and dentin are 275 and 65, respectively [48], the materials are unlikely to pose a risk of abrading the dentition. Improved material wear resistance will also reduce the risk of exposure to micro- and nano-plastic particles released from the materials. Microplastics from clear aligners have recently gained the attention of the scientific community as the potential health effects of ingested microplastics on various body systems raise concerns about patient safety [49]. Grymak et al. [50] evaluated the wear resistance of several occlusal splint materials based on manufacturing technique. In that study, milled polymethyl methacrylate (PMMA) occlusal splint material exhibited the highest wear resistance, followed by heat-cured PMMA. The 3D-printed materials showed significantly lower wear resistance than the milled and heat-cured materials. They also evaluated the wear resistance of the Splint 2.0 material, suggesting it would be preferred for patients without severe bruxing episodes.

Bacterial adhesion to surfaces generally increases with increasing average surface roughness (Ra). Limited colonization is achieved when Ra values are in the interval 0.5−1.5 μm [51, 52]. Bollen et al. [28] reported that Ra values < 0.2 µm did not further reduce the biofilm accumulation. This specific threshold value has commonly been cited for dental materials, below which no further biofilm reduction can be achieved. In our study, a thorough polishing protocol was applied to all workflows, achieving mean Ra values < 0.2 µm. This underscores the importance of a strict polishing protocol regime as a preventive measure against bacterial adhesion. However, polishing the interior parts of an occlusal device is contraindicated, leaving a significant portion of its total area unpolished [53]. We showed that print workflows without grind and polish have average Ra values of 2.09–4.52 µm (Table 4). In a study by Quirynen et al. [54], they demonstrated that increasing the surface roughness of resin strip specimens to Ra = 2.2 µm resulted in a marked increase in bacterial colonization compared with smooth strips (Ra = 0.1 µm) in the oral cavity. Shim et al. [55] reported differences in average surface roughness for resin-printed materials depending on the orientation of the object on the build plate. A 45° orientation showed the highest Ra values. The interior parts of occlusal devices may also experience different oral environmental conditions compared to the external parts. The interior parts may be in a static environment with reduced saliva flow and increased protection against shear stresses. This environment will most likely favor the growth of pathogenic species such as *S. mutans*. Wuersching et al. [56] demonstrated, using the crystal violet (CV) staining technique for biofilm quantification on occlusal devices, that biofilm, regardless of the 3D-printing material, accumulated primarily at the edges and on the inside of the devices.

To our knowledge, there is currently no widely accepted, single approach to quantifying biofilm production on dental splints. In the present study, both a static culture-plate model (which may better represent areas of the oral cavity with low shear forces) and a bioreactor-based approach with stirring (which mimics areas with higher shear forces) were employed. The 24-hour bioreactor approach showed no significant differences in biofilm mass on the materials. However, despite the large within-group variation, the Therapon workflow was the only workflow that formed significantly more biofilm in the 72-hour culture plate approach compared to the PalaXtreme workflow. The discrepancy in the methodological approaches might be explicable by the bioreactor creating sufficient shear forces to counteract bacterial attachment and subsequent biofilm formation. Both models have characteristic advantages and disadvantages with respect to simulating conditions prevalent in different regions of the oral cavity, and our results underpin the importance of using multiple approaches in the investigation of biofilm formation. Regarding differences in biofilm formation between Therapon and PalaXtreme, acrylic-based surfaces generally exhibit low SFEs, resulting in hydrophobicity and poor wettability. Materials with low SFEs have also been shown to be more resistant to bacterial adhesion compared to surfaces with higher SFEs [29, 57]. In the previously mentioned study by Quirynen et al. [54], they further concluded that changes in SFE of the tested resins had little impact on bacterial adhesion and colonization. In our study, no significant differences in SFEs across workflows were observed, making it challenging to pinpoint how SFE specifically contributes to bacterial adherence and growth. In this respect, Øilo and Bakken [26] indicate that multiple factors are likely to act simultaneously and that comparisons across studies and among materials are not straightforward. Alterations in surface roughness will most likely also alter the surface energy. Therefore, it is difficult to distinguish between the two factors [26]. It seems, however, that surface roughness is generally predominant in the context of bacterial adhesion and formation on dental materials than SFE [24, 54]. According to Hahnel et al. [58], *S. mutans* adhesion to acrylic-based materials appears to differ depending on the type of monomer mixture used, and a correlation between substratum SFE and streptococcal adhesion could not be established.

In the oral cavity, bacteria adhere to the acquired pellicle via multiple physicochemical interactions; however, the surface properties of dental materials that affect salivary pellicle formation and bacterial attachment are understudied [59]. Considering this and the *in vitro* challenges in achieving a standardized acquired enamel pellicle (AEP), this variable was excluded from our study. *In vitro* studies demonstrate considerable differences in experimental pellicles formed on dental biomaterials, while *in situ* pellicles seem to be relatively similar [29]. The high levels of proteins and other nutrients in the BHI broth medium could serve as a source for the formation of a conditional film.

In a recent review [60] on the effectiveness of post-processing for biocompatible resin-based 3D printing materials in prosthodontics, the authors highlight the importance of following the manufacturer’s protocol and note that enhancing post-treatment (especially post-photopolymerization time, rinsing time, and heat treatment) seems to improve the quality and safety of prostheses. However, due to the heterogeneity of materials and methods, it was not possible to identify an ideal post-processing protocol. Understanding the multifactorial and complex mechanisms of bacterial adhesion and growth on abiotic substrates is an important consideration for the future engineering of custom-made dental devices, tailoring material composition, design, and production methods to the benefit of patients.

Lastly, it should be noted that the experimental environment in *in vitro* studies cannot fully reproduce the complexity of intraoral conditions. A limited number of replicating certain experiments in this study may affect statistical power; findings should be interpreted with appropriate caution.

## Conclusions

Within the limitations of this study, the properties of acrylic-based occlusal devices are affected by both material chemistry and manufacturing workflow. The Therapon (mill) and PalaXtreme (autopolymer) materials showed superior water-solubility resistance, indicating enhanced material integrity and biocompatibility. The 3D-print materials demonstrated high water sorption variability and reduced hardness, which can limit their durability and suitability for high‑load clinical situations such as bruxism treatment. All workflows achieved low surface roughness, suggesting that an effective polishing protocol can produce clinically acceptable surfaces regardless of material or workflow. Comparable SFE values, including surface roughness, may explain the similarities in biofilm formation on the material surfaces, although PalaXtreme exhibited the lowest and Therapon the highest biofilm accumulation over 72 hours.

Choosing acrylic-based occlusal device materials involves balancing mechanical durability, physicochemical stability, and biological performance. Future research should focus on optimization of post‑processing and material formulations, particularly for 3D‑printed occlusal devices.

## Supplementary Material





## Data Availability

The data supporting the findings of this study are available from the corresponding author upon reasonable request.
