# Supplementary material for: Acrylic-based occlusal device materials – the influence of manufacturing techniques on material properties and the propensity for biofilm formation
Source: Biomater Investig Dent. 2026 Apr 24;13:45909. doi: 10.2340/biid.v13.45909 (PMC13127246; doi:10.2340/biid.v13.45909)
Supplement: Supplementary file 1 [file BIiD-13-45909-s1.pdf]

Supplementary material has been published as submitted. It has not been copyedited or typeset by Biomaterial Investigations in Dentistry.

Supplemental Online Material

Image of Specimen Designs

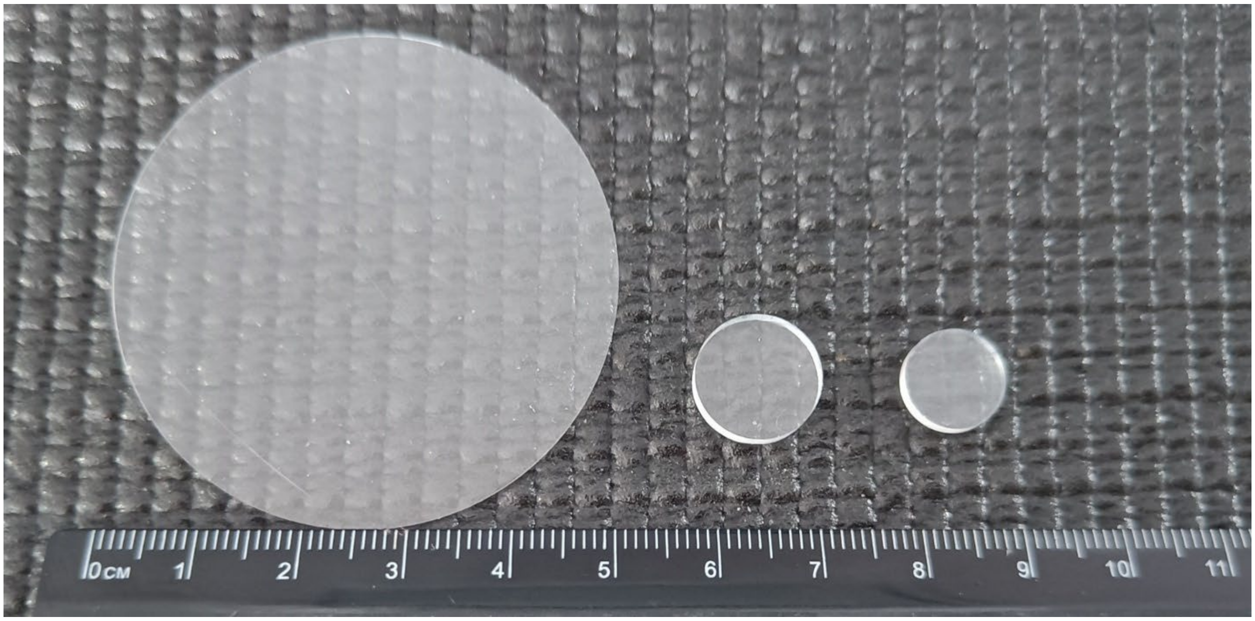

Figure S1. Different specimen designs. Left: A specimen used in the water sorption and solubility test. Middle: A specimen used in the bioreactor. Right: A specimen used in the Vickers hardness test, surface free energy, surface roughness, or the 72-h biofilm culture plate approach.

Calculation of Surface Free Energy (SFE)

Surface free energy was calculated using the Owens-Wendt-Rabel-Kaelble (OWRK) method, which separates SFE into its dispersive and polar components. SFE was determined from contact angle measurements of two test liquids following the Krüss OWRK model. The relationship is given by:

$$\gamma_L(1+\cos\,\theta) = 2\left(\sqrt{\gamma_S^d\gamma_L^d}+\sqrt{\gamma_S^p\gamma_L^p}\right)$$

where γ_S^d and γ_S^p are the dispersive and polar components of the solid surface free energy, γ_L^d and γ_L^p are the corresponding liquid components, and θ is the measured contact angle. A linear fit of the transformed equation yields the SFE components [1].

Image of the Bioreactor

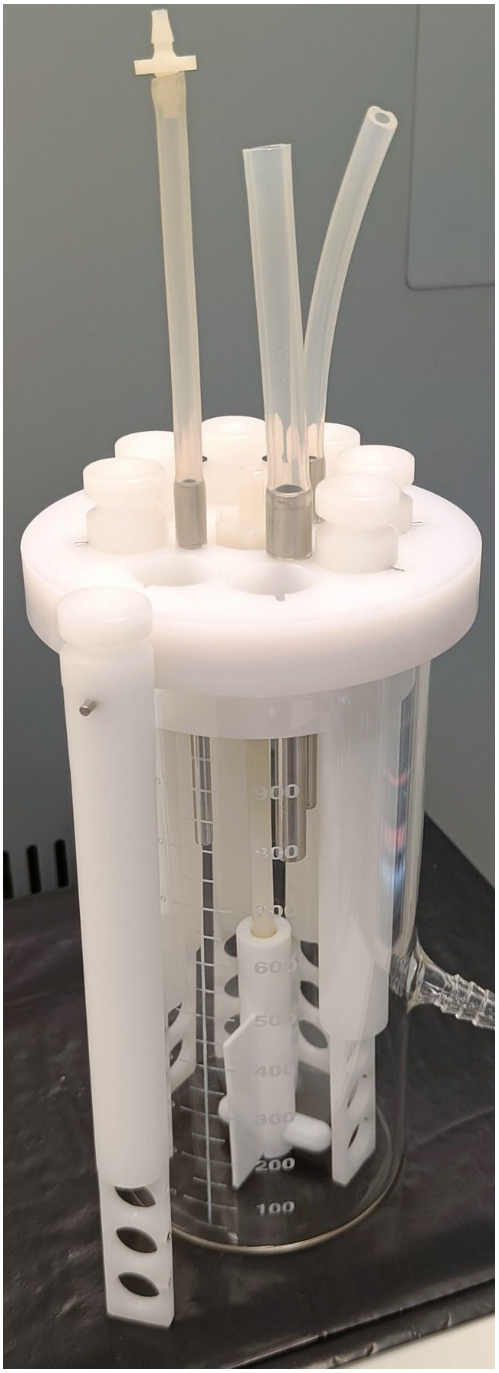

Figure S2. The CDC bioreactor. Three disc-shaped specimens can be mounted on each specimen holder. A magnetically driven stir bar is mounted on a bar in the reactor vessel.

Overview of multiplicity-adjusted p-values from all tests

- Excel file: P-values.xlsx

REFERENCES

1. Kwok DY, Neumann AW. Contact angle measurement and contact angle interpretation. Adv Colloid Interface Sci. 1999;81(3):167-249.
