# Supplementary material for: Acrylic-based occlusal device materials – the influence of manufacturing techniques on material properties and the propensity for biofilm formation
Source: Biomater Investig Dent. 2026 Apr 24;13:45909. doi: 10.2340/biid.v13.45909 (PMC13127246; doi:10.2340/biid.v13.45909)
Supplement: Supplementary file 2 [file BIiD-13-45909-s2.pdf]

Supplementary material has been published as submitted. It has not been copyedited or typeset by Biomaterial Investigations in Dentistry.

Overview of adjusted multiplicity-adjusted p-values from the experiments

Sorption and solubility test (ISO 20795-2:2013)

Sorption

Dunn's multiple comparisons test	Significant?	**P value**
Splint 2.0 - AF vs. Splint 2.0 - OF	No	>0.9999
Splint 2.0 - AF vs. LT Clear - FC	Yes	0.0037
Splint 2.0 - AF vs. LT Clear - OF	Yes	0.0056
Splint 2.0 - AF vs. Therapon	No	>0.9999
Splint 2.0 - AF vs. PalaXtreme	No	0.3522
Splint 2.0 - OF vs. LT Clear - FC	Yes	0.0042
Splint 2.0 - OF vs. LT Clear - OF	Yes	0.0064
Splint 2.0 - OF vs. Therapon	No	>0.9999
Splint 2.0 - OF vs. PalaXtreme	No	0.3866
LT Clear - FC vs. LT Clear - OF	No	>0.9999
LT Clear - FC vs. Therapon	No	0.3204
LT Clear - FC vs. PalaXtreme	No	>0.9999
LT Clear - OF vs. Therapon	No	0.4239
LT Clear - OF vs. PalaXtreme	No	>0.9999
Therapon vs. PalaXtreme	No	>0.9999

Solubility

Dunn's multiple comparisons test	Significant?	**P value**
Splint 2.0 - AF vs. Splint 2.0 - OF	No	>0.9999
Splint 2.0 - AF vs. LT Clear - FC	No	>0.9999
Splint 2.0 - AF vs. LT Clear - OF	No	>0.9999
Splint 2.0 - AF vs. Therapon	No	0.2077
Splint 2.0 - AF vs. PalaXtreme	No	>0.9999
Splint 2.0 - OF vs. LT Clear - FC	No	>0.9999
Splint 2.0 - OF vs. LT Clear - OF	No	0.426
Splint 2.0 - OF vs. Therapon	No	0.5335
Splint 2.0 - OF vs. PalaXtreme	No	>0.9999

LT Clear - FC vs. LT Clear - OF	No	>0.9999
LT Clear - FC vs. Therapon	Yes	0.006
LT Clear - FC vs. PalaXtreme	No	0.153
LT Clear - OF vs. Therapon	Yes	0.0003
LT Clear - OF vs. PalaXtreme	Yes	0.0133
Therapon vs. PalaXtreme	No	>0.9999

Vickers hardness test

Tukey's multiple comparisons test	Significant?	**P value**
Splint 2.0 - AF vs. Splint 2.0 - OF	No	0.2659
Splint 2.0 - AF vs. LT Clear - FC	Yes	0.0003
Splint 2.0 - AF vs. LT Clear - OF	Yes	<0.0001
Splint 2.0 - AF vs. Therapon	Yes	<0.0001
Splint 2.0 - AF vs. PalaXtreme	Yes	<0.0001
Splint 2.0 - OF vs. LT Clear - FC	Yes	<0.0001
Splint 2.0 - OF vs. LT Clear - OF	Yes	<0.0001
Splint 2.0 - OF vs. Therapon	Yes	<0.0001
Splint 2.0 - OF vs. PalaXtreme	Yes	<0.0001
LT Clear - FC vs. LT Clear - OF	No	0.4094
LT Clear - FC vs. Therapon	Yes	<0.0001
LT Clear - FC vs. PalaXtreme	Yes	<0.0001
LT Clear - OF vs. Therapon	Yes	<0.0001
LT Clear - OF vs. PalaXtreme	Yes	<0.0001
Therapon vs. PalaXtreme	Yes	<0.0001

**Surface free energy (SFE)**	Significant?	**P value**
Tukey's multiple comparisons test		
Therapon vs. PalaXtreme	No	0.4555
Therapon vs. LT Clear - FC	No	0.0896
Therapon vs. LT Clear - OF	No	0.2042
Therapon vs. Splint 2.0 - AF	No	0.3231
Therapon vs. Splint 2.0 - OF	No	0.2709
PalaXtreme vs. LT Clear - FC	No	0.8624

PalaXtreme vs. LT Clear - OF	No	0.9891
PalaXtreme vs. Splint 2.0 - AF	No	0.9997
PalaXtreme vs. Splint 2.0 - OF	No	0.9983
LT Clear - FC vs. LT Clear - OF	No	0.994
LT Clear - FC vs. Splint 2.0 - AF	No	0.9516
LT Clear - FC vs. Splint 2.0 - OF	No	0.9754
LT Clear - OF vs. Splint 2.0 - AF	No	0.9994
LT Clear - OF vs. Splint 2.0 - OF	No	>0.9999
Splint 2.0 - AF vs. Splint 2.0 - OF	No	>0.9999

**Surface roughness (Ra)**	Significant?	**P value**
Tukey's multiple comparisons test		
Therapon vs. PalaXtreme	No	0.5996
Therapon vs. LT Clear - FC	No	0.9272
Therapon vs. LT Clear - OF	No	0.8924
Therapon vs. Splint 2.0 - AF	No	0.7749
Therapon vs. Splint 2.0 - OF	No	0.9648
PalaXtreme vs. LT Clear - FC	No	0.9808
PalaXtreme vs. LT Clear - OF	No	0.9908
PalaXtreme vs. Splint 2.0 - AF	No	0.9995
PalaXtreme vs. Splint 2.0 - OF	No	0.9542
LT Clear - FC vs. LT Clear - OF	No	>0.9999
LT Clear - FC vs. Splint 2.0 - AF	No	0.9989
LT Clear - FC vs. Splint 2.0 - OF	No	>0.9999
LT Clear - OF vs. Splint 2.0 - AF	No	0.9998
LT Clear - OF vs. Splint 2.0 - OF	No	0.9998
Splint 2.0 - AF vs. Splint 2.0 - OF	No	0.9941

**Surface roughness (Ra) - non ground and polished**	Significant?	**P value**
Tukey's multiple comparisons test		
LT Clear - FC vs. LT Clear - OF	No	0.9504
LT Clear - FC vs. Splint 2.0 - AF	Yes	0.0126
LT Clear - FC vs. Splint 2.0 - OF	No	0.3914
LT Clear - OF vs. Splint 2.0 - AF	Yes	0.0255
LT Clear - OF vs. Splint 2.0 - OF	No	0.6684
Splint 2.0 - AF vs. Splint 2.0 - OF	No	0.1287

**24-h bioreactor approach**	Significant?	**P value**
Tukey's multiple comparisons test		
Therapon vs. PalaXtreme	No	>0.9999
Therapon vs. LT Clear - FC	No	0.9838
Therapon vs. LT Clear - OF	No	0.9155

**72-h culture plate approach**	Significant?	**P value**
Tukey's multiple comparisons test		
Therapon vs. PalaXtreme	Yes	0.0452
Therapon vs. LT Clear - FC	No	0.1216
Therapon vs. LT Clear - OF	No	0.1108

Therapon vs. Splint 2.0 - AF	No	0.9505
Therapon vs. Splint 2.0 - OF	No	0.8655
PalaXtreme vs. LT Clear - FC	No	0.9921
PalaXtreme vs. LT Clear - OF	No	0.9433
PalaXtreme vs. Splint 2.0 - AF	No	0.9698
PalaXtreme vs. Splint 2.0 - OF	No	0.9025
LT Clear - FC vs. LT Clear - OF	No	0.9994
LT Clear - FC vs. Splint 2.0 - AF	No	>0.9999
LT Clear - FC vs. Splint 2.0 - OF	No	0.9967
LT Clear - OF vs. Splint 2.0 - AF	No	>0.9999
LT Clear - OF vs. Splint 2.0 - OF	No	>0.9999
Splint 2.0 - AF vs. Splint 2.0 - OF	No	0.9998

Therapon vs. Splint 2.0 - AF	No	0.0631
Therapon vs. Splint 2.0 - OF	No	0.0518
PalaXtreme vs. LT Clear - FC	No	0.9889
PalaXtreme vs. LT Clear - OF	No	0.993
PalaXtreme vs. Splint 2.0 - AF	No	>0.9999
PalaXtreme vs. Splint 2.0 - OF	No	>0.9999
LT Clear - FC vs. LT Clear - OF	No	>0.9999
LT Clear - FC vs. Splint 2.0 - AF	No	0.9983
LT Clear - FC vs. Splint 2.0 - OF	No	0.9942
LT Clear - OF vs. Splint 2.0 - AF	No	0.9992
LT Clear - OF vs. Splint 2.0 - OF	No	0.9967
Splint 2.0 - AF vs. Splint 2.0 - OF	No	>0.9999
